# Enzymatic Conversion of Different Qualities of Refined Softwood Hemicellulose Recovered from Spent Sulfite Liquor

**DOI:** 10.3390/molecules27103207

**Published:** 2022-05-17

**Authors:** Abhishek Bhattacharya, Samuel Butler, Basel Al-Rudainy, Ola Wallberg, Henrik Stålbrand

**Affiliations:** 1 Biochemistry and Structural Biology, Department of Chemistry, Lund University, P.O. Box 124, 22100 Lund, Sweden; abhishek.bhattacharya@biochemistry.lu.se (A.B.); samuel.john.butler@gmail.com (S.B.); 2 Department of Chemical Engineering, Lund University, P.O. Box 124, 22100 Lund, Sweden; basel.al-rudainy@chemeng.lth.se (B.A.-R.); ola.wallberg@chemeng.lth.se (O.W.)

**Keywords:** spent sulfite liquor, pre-treatment and purification, lignin, acetylated galactoglucomannan, β-mannanase, *Tr*Man5A, Viscozyme L, bioconversion

## Abstract

Spent sulfite liquor (SSL) from softwood processing is rich in hemicellulose (acetyl galactoglucomannan, AcGGM), lignin, and lignin-derived compounds. We investigated the effect of sequential AcGGM purification on the enzymatic bioconversion of AcGGM. SSL was processed through three consecutive purification steps (membrane filtration, precipitation, and adsorption) to obtain AcGGM with increasing purity. Significant reduction (~99%) in lignin content and modest loss (~18%) of polysaccharides was observed during purification from the least pure preparation (UFR), obtained by membrane filtration, compared to the purest preparation (AD), obtained by adsorption. AcGGM (~14.5 kDa) was the major polysaccharide in the preparations; its enzymatic hydrolysis was assessed by reducing sugar and high-performance anion-exchange chromatography analysis. The hydrolysis of the UFR preparation with Viscozyme L or *Trichoderma reesei* β-mannanase *Tr*Man5A (1 mg/mL) resulted in less than ~50% bioconversion of AcGGM. The AcGGM in the AD preparation was hydrolyzed to a higher degree (~67% with *Tr*Man5A and 80% with Viscozyme L) and showed the highest conversion rate. This indicates that SSL contains enzyme-inhibitory compounds (e.g., lignin and lignin-derived compounds such as lignosulfonates) which were successfully removed.

## 1. Introduction

Environmental issues such as climate change have drawn worldwide attention to alternative non-fossil resources. Of interest are new, sustainable, eco-friendly, and cost-effective routes for producing energy carriers and chemicals [1]. Among natural resources, plant polysaccharides hold potential as extremely abundant, renewable, biocompatible, and non-toxic raw materials [2]. With a focus on cellulose saccharification, significant efforts have been made to ameliorate the enzymatic digestibility of recalcitrant lignocellulosic raw materials for the production of biofuels and chemicals [3]. Furthermore, a holistic approach has recently emerged that focuses on increased utilization of all plant cell-wall components, and parts of the forestry industry are being transformed into bio-refineries, including upgrading of side streams to useful products [2,4,5]. Traditionally, the organic content of many of these streams is used for heat and power generation. However, a sustainable forest bio-refinery concept would likely benefit from valorizing the underutilized residual components of wood, such as hemicellulose and lignin, by developing value-added by-products [6,7,8]. Hemicelluloses such as xylan and β-mannans are integral parts of secondary cell walls and are associated with cellulose and lignin [9,10].

In chemical pulping methods such as kraft and sulfite processes, a major part of the hemicelluloses is solubilized, partially decomposed, and released in side-streams including black liquor and spent sulfite liquor (SSL), in which they form a significant constituent, together with soluble lignin, lignin derivatives, and low molecular weight compounds [11]. Acetyl galactoglucomannan (AcGGM) is the dominant hemicellulose in softwoods such as Scots pine and Norway spruce, constituting approximately 20% of the dry wood matter [12]. AcGGM has a mannosyl (β-1,4-Man) and glucosyl (β-1,4-Glu) backbone. The mannosyl units can be substituted with α-1,6-linked galactosyl units (α-1,6-Gal) and partially substituted by O-acetyl groups at the C2 and C3 positions [12,13]. Recently, the utilization of AcGGM has drawn much attention. Polymeric AcGGM has been studied for barrier film formation [14] and has been chemically modified for the development of a variety of materials including hydrogels [15,16] and non-ionic polymeric surfactants [17]. Monosugars originating from hemicelluloses such as AcGGM and present in SSL have also been used for the microbial production of polyhydroxybutyrate, a polymer with material applications [18]. The enzymatic hydrolysis of β-mannans into monosugars followed by the production of bioethanol or chemicals is a possible valorization route [19]. Recent developments in enzymatic bioconversion techniques of β-mannans into novel functional glycosides such as alkyl glycosides, allyl glycosides, and glycosyl-methylacrylates [20,21,22], and into potential prebiotic oligosaccharides [23,24], offer additional new opportunities for the enzyme-based valorization of AcGGM from side streams.

Softwood-based SSL contains AcGGM and its degradation products, but is also rich in lignin and lignin-derived compounds such as lignosulfonates (LS) [25,26,27]. It is known that the presence of lignin results in lower enzymatic cellulose saccharification yields due to steric hindrance, non-productive binding of cellulases onto lignin, and enzyme deactivation [28,29]. The current understanding of the mechanism involved in enzyme-lignin interaction is largely based on the interaction of insoluble lignin with cellulases [30,31]. However, the interaction of water-soluble lignin, particularly LS, with enzyme(s) and their potential enzyme inhibitory role has not been explored in detail [29,32].

Although the effect of lignin and lignin derivatives on enzymatic cellulose conversion is well studied [30,33,34], very few studies have investigated their effect on mannanolytic enzymes [35]. The use of commercial cocktails like Viscozyme L, Celluclast 1.5 L, and Cellic CTec2 for enzymatic hydrolysis of lignocellulosic biomass is well documented [36,37]. However, the potential of such enzyme cocktails for the conversion of the β-mannans present in industrial side-streams (such as SSL) is an interesting topic that merits greater attention.

To explore the acetylated galactoglucomannan obtained from spent sulfite liquor (SSL-AcGGM) as a raw material for valorization, it is important to investigate the effect of its purification on the efficiency of enzymatic bioconversion. The importance of the concentration and purification of AcGGM from dilute waste streams has been emphasized previously [15,26,38,39]. However, depending on which end-product is targeted, the optimal degree of purity may vary, not only for direct material applications but also when enzymatic and/or microbial bioconversion is involved. This study focuses on the first steps of enzyme-aided valorization of SSL-AcGGM. The effect of the purity of AcGGM on enzymatic conversion efficacy is assessed.

With SSL as the starting material, several AcGGM-containing preparations with different purities and lignin contents were obtained through consecutive purification procedures. Well-characterized AcGGM from thermomechanical pulping (TMP-AcGGM) [40,41] was included as reference material. After comparing several commercial enzyme cocktails, Viscozyme L was selected for bioconversion of the different SSL-AcGGM preparations. In addition, the β-mannanase *Tr*Man5A from the industrially important enzyme-producing fungus *Trichoderma reesei* [42] was selected to gain knowledge on bioconversion by a well-defined mono-component enzyme. Thus, the current investigation increases our understanding of enzymatic hydrolysis and the bioconversion of AcGGM for which different end-products and purity requirements can be envisioned.

## 2. Results and Discussion

### 2.1. Characterization of Acetyl-Galactoglucomannan from Spent Sulfite Liquor

#### 2.1.1. Compositional Analysis

The AcGGM used in this study originated from the industrial waste stream SSL. The retentate from an on-site ultrafiltration device (SSL concentrate) was further concentrated using membrane filtration [25]; this preparation was termed ultrafiltration retentate (UFR). The UFR preparation was purified through precipitation using acetone as the antisolvent [16]; this preparation was designated as antisolvent-precipitate concentrate (AC). The AC preparation was further purified by two different polymeric resins in series [27]; the final column permeate was termed adsorption preparation (AD). The concentration and purification procedures are described in Section 3.2. The composition of the various preparations is shown in Table 1.

The major compounds found in the various preparations were mainly lignin and polysaccharides. As seen in Table 1, the major polysaccharide in all three preparations was galactoglucomannan (GGM), with a concentration of 43.89, 39.94 and, 36.29 mg/g, for UFR, AC, and AD, respectively, corresponding to 92.3, 93.3, and 93.5% of the glycans in the preparations. Lignin was the main component found in the UFR preparation (192.2 mg/g, corresponding to 80.1% of the dry weight). The removal of the lignin from the solution using anti-solvent precipitation was effective. The lignin concentration decreased to 25.4 mg/g in the AC preparation, resulting in ~87% removal. The adsorption process was also effective at removing the remaining lignin. The concentration of lignin decreased about ten-fold from 25.4 mg/g in AC to 2.6 mg/g in the AD preparation, giving 98.6% lignin removal compared to the UFR preparation while retaining 81.7% of the polysaccharides (Table 1). The concentration of GGM in the solutions did not vary by a large margin, with a total loss of 17.3% from the ultrafiltration retentate to the preparation after adsorption chromatography. The purity of the GGM in the preparations was expressed as % of dry weight (with total lignin but excluding ash) in increasing order from UFR (18.3%), AC (58.5%), and AD (87.3%). Furthermore, the yield of GGM for the antisolvent precipitation (91.0%) and the adsorption (90.8%) purification steps were appreciably higher compared to the membrane filtration (62.1%). These results highlighted the effectiveness of lignin removal and the high selectivity of these separation methods toward the removal of lignin. The acetyl content was determined to be 1.40 ± 0.03, 1.23 ± 0.03, and 1.10 ± 0.04 mg/g in the UFR, AC, and AD preparations, respectively. The molar composition of mannosyl: galactosyl: glucosyl: acetyl content in UFR (1.0:0.38:0.34:0.21), AC (1.0:0.38:0.32:0.20) and AD (1.0:0.37:0.31:0.19) did not vary significantly. The interpreted composition of AcGGM in the UFR and AC preparations had a similar composition as AcGGM prepared previously in a similar way from SSL of the same process [39], but the acetyl content was ~2.6 fold lower in the current study. Furthermore, 1.3 times less xylan was observed. These rather modest differences are not surprising, since variations in the raw material and industrial process conditions likely influence the composition of the SSL and its components [25,27,39]. Variations in the molar composition of hemicelluloses in industrial side streams have also been observed in other cases [13,40]. Hemicellulose and side-stream characterization of the kind carried out in the present study will likely contribute to overcoming feedstock and side-stream heterogeneity as a possible hurdle in the bioeconomy [43].

#### 2.1.2. Apparent Molecular Weight of Acetyl-Galactoglucomannan in the Preparations

**Figure 1:**
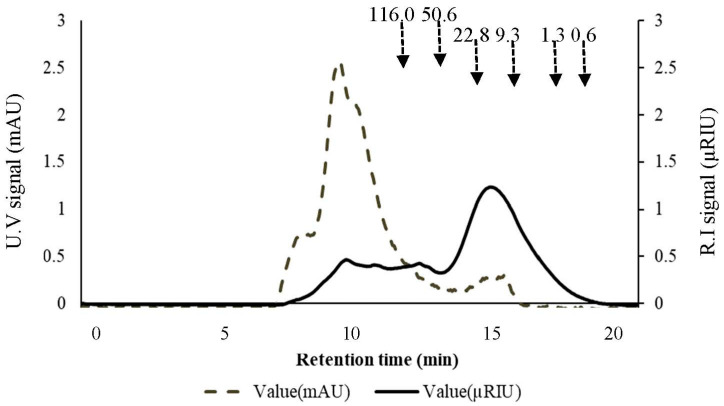
Size exclusion chromatography of adsorption preparation (AD). The analytical separation was achieved using Shodex, SB-803-HQ column (Shodex, Tokyo, Japan) with a variable wavelength UV detector (mAU) at 280 nm (Thermo Fisher, Waltham, MA, USA) and RI 101 detector (µRIU) (Shodex, Tokyo, Japan). Water was used as an eluent at a flow rate of 0.5 mL/min, the substrate was used at 1 mg/mL of GGM, and the temperature was maintained at 25 °C. Arrows (dashed) mark the peak maximum of pullulan standards (kDa). The RI peak maximum (solid arrow) was used to estimate the apparent molecular weight of the AcGGM product, based on the logarithmic calibration curve of the pullulan standards.

To determine the polymeric and/or oligomeric nature of the soluble AcGGM and to further assess the purity of the different preparations of acetyl-galactoglucomannan from SSL (SSL-AcGGM), size exclusion chromatography (SEC) analysis was carried out (Figure 1 and Figure S1). Since lignin (and derivates thereof) and AcGGM were the major components in the preparations (Table 1); the elution profiles based on ultraviolet (UV) and refractive index (RI) detection can be used to distinguish between, and estimate the size of, such molecules. Based on the purest preparation (AD), the apparent molecular weight (Mw) of the peak maximum corresponding to AcGGM was estimated to be ~14.5 kDa using pullulan standards (Figure 1). Since this prominent AcGGM product eluted with a similar retention time in all the preparations (Figure S1a), we suggest that the AcGGM from SSL in the different preparations was polymeric with an apparent Mw around 14.5 kDa. A previous study, where the AcGGM obtained from SSL was processed similarly to that in the present study and was similar in composition, reported the apparent Mw of the AcGGM with a peak maximum of ~10.0 kDa [16] using an analogous SEC-procedure. The variations in the reported apparent molecular weight determined with similar methods will undoubtedly be impacted by the feedstock and, in particular, its processing or pre-treatment [44,45].

In this study, we observed a significant reduction in the SEC UV signal in the AC and AD preparations compared to the UFR preparation (Figure S1b). This indicated the removal of lignin (and/or its derivatives), which was corroborated by compositional analyses (Table 1). Some overlap between the UV and RI signals was consistent in all three preparations, considering the reduced lignin content in the AC and AD preparations (Figure 1 and Figure S1). This may have been due to the presence of lignin-carbohydrate complexes (LCC), which have previously been observed in softwood SSL-AcGGM preparations [25,39].

Since the preparations were remarkably similar in AcGGM composition and content but differed in their lignin content (Table 1), these preparations were suitable for assessing the effect of AcGGM purity on enzymatic conversion. For this, a selection of commercially available enzyme cocktails was made based on their efficacy for the hydrolysis of a well-characterized TMP-AcGGM [41], as described in the forthcoming sections.

### 2.2. Selection of Commercial Enzyme Cocktails

#### 2.2.1. Determining the Hydrolytic Efficiency of Commercial Enzyme Cocktails

Three different commercial enzyme cocktails with extensive application in lignocellulose hydrolysis [36,37] were initially selected for hydrolysis of SSL-AcGGM. These were Celluclast 1.5 L and Viscozyme L, which are derived from *T. reesei* and *Aspergillus aculeatus*, respectively, and Cellic CTec 2 which is an enzyme blend Furthermore, the well-characterized β-mannanase from *T. reesei, Tr*Man5A was also included in the study. The evaluation of the hydrolytic efficacy of these commercial cocktails was carried out using the well characterized TMP-AcGGM [41] as a substrate. The hydrolytic efficiency was monitored by incubating 0.1 mg/mL of either an enzyme cocktail or *Tr*Man5A with 100 mg/mL of GGM, at 40 °C, pH 5.0 (100 mM acetate buffer) for 24 h (Figure S2). Based on reducing sugar equivalents, the highest percentage conversion was observed with Viscozyme L (32.5% ± 1.2) followed by *Tr*Man5A (30.2% ± 1.2), Celluclast 1.5 L (18.0% ± 1.1), and CTec 2 (11.3% ± 0.6) (Figure S2). The relatively high conversion efficiency with *Tr*Man5A was expected, considering this enzyme is potent in pre-treated softwood saccharification, by attacking the AcGGM backbone [46,47].

#### 2.2.2. Enzyme Activities in Commercial Enzyme Cocktails and Enzyme Stability

To understand the high conversion achieved with Viscozyme L compared to the other two enzyme cocktails, the different enzyme activities present in the cocktails were investigated (Table S1). Viscozyme L at 0.1 mg/mL protein concentration exhibited high β-mannanase (4191.7 ± 97 U/mL) and α-galactosidase (364.1 ± 31 U/mL) activity, substantial β-glucosidase (169.7 ± 17 U/mL) but low β-mannosidase (29.6 ± 0.3 U/mL) activity. However, Celluclast 1.5 L exhibited ~10-fold lower β-mannanase and α-galactosidase activities. Also, no detectable β-mannanase activity was observed with CTec 2, while some α-galactosidase (76.1 ± 6 U/mL) activity was detected (Table S1). For *Tr*Man5A, at 0.1 mg/mL the β-mannanase activity was 2304.1 ± 57 U/mL. Based on the TMP-AcGGM hydrolysis and subsequent analysis of enzyme activities present in the three enzyme cocktails, Viscozyme L was selected for further studies along with *Tr*Man5A.

Furthermore, the stability of Viscozyme L and *Tr*Man5A in 100 mM sodium acetate buffer at pH 5.0 and 40 °C was analyzed over 48 h (Figure S3). More than 85% and ~80% residual β-mannanase, α-galactosidase, and β-glucosidase activities were retained in Viscozyme L after 24 and 48 h, respectively. *Tr*Man5A retained ~90% and ~80% residual β-mannanase activity after 24 h and 48 h respectively. This shows that both Viscozyme L and *Tr*Man5A are, at large, stable over 48 h at 40 °C and pH 5.0.

### 2.3. Effect of Purification on Hydrolysis of Acetylgalactoglucomannan from Spent Sulfite Liquor

#### 2.3.1. Hydrolysis of Different Acetyl-Galactoglucomannan Preparations Using *Tr*Man5A and Viscozyme L

**Figure 2:**
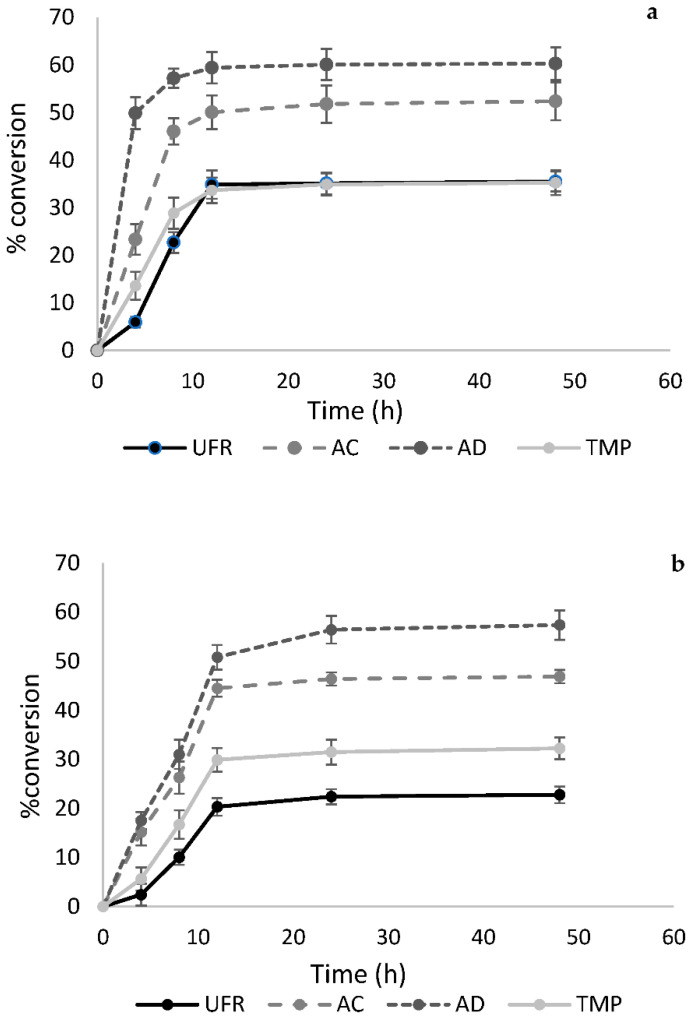
Hydrolysis of different SSL-AcGGM preparations and TMP-AcGGM using (**a**) *Tr*Man5A (**b**) Viscozyme L. UFR, AC and AD SSL-AcGGM preparations, and the TMP-AcGGM were used at an initial concentration of 100 mg/mL of GGM and both *Tr*Man5A and Viscozyme L at 0.1 mg/mL of protein (enzyme) loading. The hydrolysis reactions were carried out at 40 °C, pH 5.0 for 48 h. The reducing sugar equivalents were determined using the DNS method with a mannose standard curve and were reported as % conversion. The reactions were carried out in triplicate and mean values ± SD are presented.

To understand the effect of purification of SSL-AcGGM on enzymatic hydrolysis, the three different SSL-AcGGM preparations (in increasing order of purity: UFR, AC, and AD) were hydrolyzed using *Tr*Man5A or Viscozyme L. TMP-AcGGM was hydrolyzed in the same way. The initial substrate concentration was 100 mg/mL of GGM and the protein loading for *Tr*Man5A and Viscozyme L was 0.1 mg/mL. The hydrolysis reactions were carried out at 40 °C and pH 5.0 for 48 h, and aliquots were removed at different time points and analyzed by the reducing sugar 3-5 dinitrosalicylic acid (DNS) method; see Figure 2. The degree of conversion (%) based on reducing sugar equivalents indicated that the highest conversion was achieved for the AD preparation (60.3%), followed by AC (52.4%), UFR (35.5%), and TMP (35.3%), using *Tr*Man5A; see Figure 2a. For an endo-mannanase such as *Tr*Man5A, that is assumed to produce mainly short oligosaccharides, the approx. 60% conversion achieved for the AD preparation was a high value and would imply a significant release of mannose, as discussed in the next section. With Viscozyme L, the highest conversion was observed for AD (57.4%), followed by AC (46.8%), TMP (32.2%), and UFR (22.8%); see Figure 2b. Notably, the rate of conversion during the first hours reflected the degree of conversion at the end, i.e., the early rate of conversion was highest for AD, which also yielded the highest end-point conversion; see Figure 2. No significant change in conversion was observed after 12 h for the different preparations from SSL-AcGGM or TMP-AcGGM using either *Tr*Man5A or Viscozyme L.

#### 2.3.2. Analysis of Major Hydrolysis Products Using High-Performance Anion-Exchange Chromatography with Pulsed Amperometric Detection (HPAEC-PAD)

**Figure 3:**
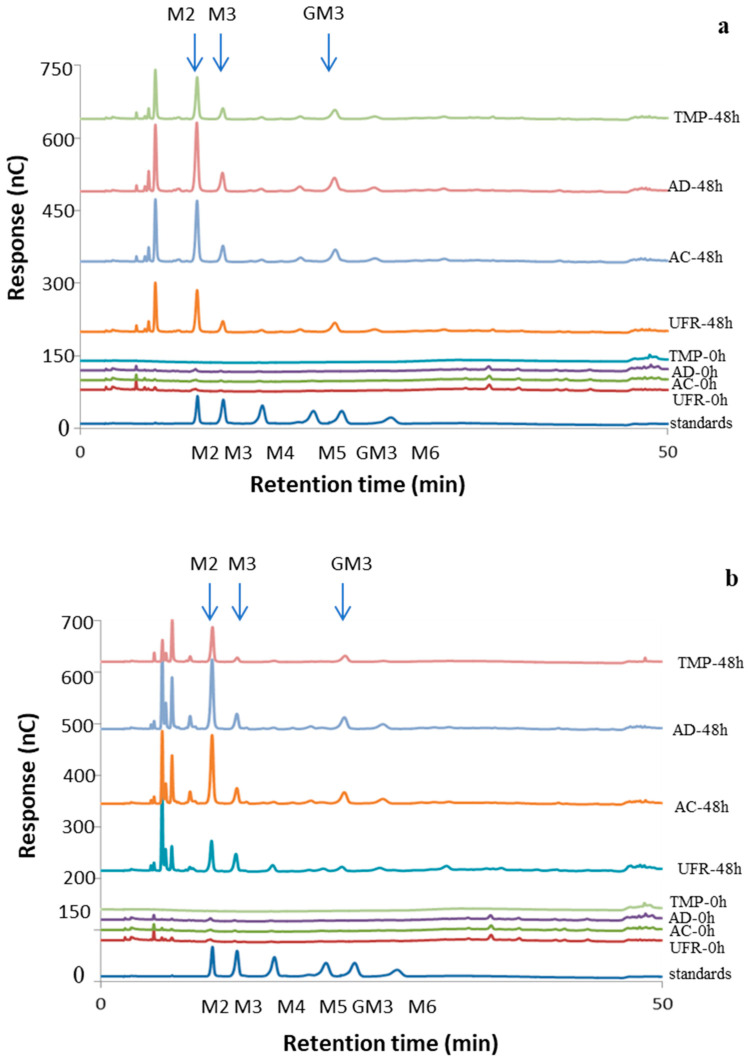
Product profile after hydrolysis of SSL-AcGGM preparations and TMP-AcGGM using (**a**) *Tr*Man5A (**b**) Viscozyme L. Aligned HPAEC-PAD chromatograms depicting the oligosaccharide product profile obtained after hydrolysis of UFR, AC, AD SSL-AcGGM preparations and TMP-AcGGM used at an initial concentration of 100 mg/mL of GGM after hydrolysis by *Tr*Man5A and Viscozyme L, used at a protein loading of 0.1 mg/mL. The enzymatic hydrolysis was carried out over 48 h of incubation at 40 °C and pH 5.0. The arrowheads indicate the major products that were determined in the hydrolysate based on their retention time with known standards. M2: mannobiose; M3: mannotriose, M4: mannotetraose; M5: mannopentaose; M6: mannohexaose; GM3: galactosyl-mannotriose.

The conversion percentage calculated based on the reducing sugar DNS analysis presented in the previous section can only detect the reducing end of a saccharide, regardless of its degree of polymerization. Consequently, 100% conversion with this method is reached only when a polysaccharide is hydrolyzed into monosaccharides. Thus, the DNS method is a quick and convenient method to estimate the degree of hydrolysis, but it gives no insight into which major hydrolysis products are formed. Therefore, the hydrolysates obtained after 8, 24, and 48 h were further analyzed for monosaccharides and oligosaccharides by HPAEC-PAD, which allowed us to take into account all monosaccharide units of the hydrolysis products when calculating the conversion percentage. The products that eluted with the retention times corresponding to the known standards were quantified. The qualitative product profiles obtained from enzymatic hydrolysis of the various AcGGM preparations were similar and the profiles obtained with Viscozyme L were similar to those of *Tr*Man5A (Figure 3). *Tr*Man5A is an endo-mannanase; it produces mannobiose and mannotriose, essentially as the only end-products from linear polymannose (water-insoluble ivory nut mannan) [42]. In accordance with this, after 48 h of incubation (Figure 3a), *Tr*Man5A released products corresponding to mannobiose, M2 (contributing with 16.5% conversion of the original substrate), mannotriose, M3 (4.7%), and galactosyl mannotriose, GM3 (8.3%) as dominant end-products from the AD preparation of SSL-AcGGM samples (Figure S4), with numeric values given in Table S2. Other products with a degree of polymerization (DP) of 4–5 were also observed (Figure 3a). Similarly, products corresponding to M2 (12.7%), M3 (2.0%), and GM3 (3.1%) were quantified as major oligosaccharide products from TMP-AcGGM hydrolysis (Figure 3a and Figure S4). Interestingly, comparably high amounts of mannose (5.1–10.8%) were released by *Tr*Man5A (Figure S4), which explains how 60% conversion could be reached by the DNS method with the AD preparation (Section 2.3.1) (Figure 2a). Although β-mannosidase activity is present in glycoside hydrolase family 5 subfamily 7 (GH5-7) [48], no significant β-mannosidase activity has been detected in purified preparations of *Tr*Man5A [42] and was confirmed by us (Table S1). The concentration of M3 was lower at 48 h than at 8 h (Figure S4), indicating its hydrolysis and explaining at least part of the mannose release. A previous report on the hydrolytic action of *Tr*Man5A on AcGGM also reported minor mannose release [47]. Our study further underlines that the type of mannan substrate used may influence the mannose release.

The major oligosaccharide products after 48 h hydrolysis (Figure 3b) of AD using Viscozyme L were M2 (contributing with 14.7% conversion of the original substrate), M3 (6.9%), and GM3 (6.6%) (Figure S5); the numeric values are presented in Table S3. Also, M2 (10.4%), M3 (1.8%), and GM3 (4.2%) were quantified as major oligosaccharide products from TMP-AcGGM hydrolysis (Figure S5). Viscozyme L is an enzyme cocktail derived from an *A. aculeatus* strain [49]. Furthermore, *A. aculeatus* produces a β-mannanase that is homologous to *Tr*Man5A (58% protein identity) and produces mannobiose, mannotriose, and mannose as the major hydrolysis products from ivory nut mannan [42,50]. This provides a plausible explanation for the similar mannose and mannan-oligosaccharide profile observed for *Tr*Man5A and Viscozyme L, even though this cocktail exhibited rather low levels of β-mannosidase activity (Table S1). The galactose and glucose release (Figure S5) could be attributed to the substantial α-galactosidase and β-glucosidase activities, respectively, present in Viscozyme L (Table S1). Viscozyme L has been used in the hydrolysis of lignocellulosic biomass [51] and pectinaceous fibers [49]. Here, we show its applicability for the hydrolysis of AcGGM-enriched softwood bio-refinery side streams, for which it, in comparison, seems to have good potential (Figure S2) in terms of matching enzyme specificities (Table S1). Several other enzyme cocktails (e.g., Cellic CTec 3, Celluclast 1.5 L, and Carezyme 1000 L) have been studied for softwood hydrolysis, but often with a focus on efficient cellulose conversion and, when measured, displayed more limited mannan conversion [46,52].

#### 2.3.3. Degree of Conversion of the SSL-AcGGM Preparations

**Figure 4:**
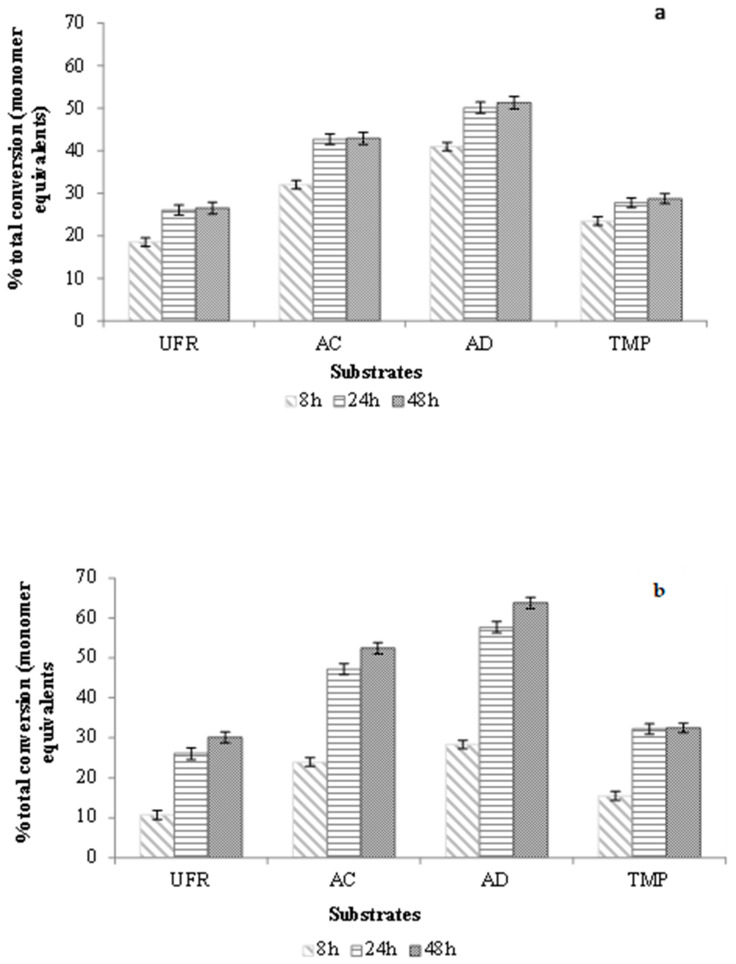
Quantitative estimation of products from different SSL-AcGGM preparations and TMP-AcGGM (**a**) *Tr*Man5A (**b**) Viscozyme L. All the substrates were used at an initial concentration of 100 mg/mL of GGM and the enzyme concentration for both *Tr*Man5A and Viscozyme L was at 0.1 mg/mL. The enzymatic hydrolysis was carried out over 48 h of incubation at 40 °C and pH 5.0. The mono- and oligo-saccharide analysis was carried out using HPAEC-PAD with CarboPac PA20 and PA200 columns, respectively. The quantities of quantifiable monosaccharides and oligosaccharides after enzymatic hydrolysis was reported as % conversion (sum of produced oligosaccharides as monomer equivalents and mono-saccharides/total polymeric galactosyl, glucosyl, and mannosyl content of the original substrate in monomer equivalents). The values are based on the data presented in Figures S4 and S5 with numeric values given in Tables S2 and S3.

Based on the HPAEC-PAD product analysis presented in the previous section, the quantification of released hexoses and quantifiable mannan-oligosaccharides (in terms of saccharide monomer equivalents) was compared to the monomer equivalents of GGM and the degree of conversion was reported as % total conversion (Figure 4). The highest conversion was achieved with the most purified preparation (AD) with *Tr*Man5A (51.3% ± 1.4) as well as with Viscozyme L (63.8% ± 1.4) after 48 h of incubation, followed by the AC (42.9% ± 1.5 with *Tr*Man5A; 52.4% ± 1.3 with Viscozyme L) and UFR (26.5% ± 1.2 with *Tr*Man5A; 30.1% ± 1.3 with Viscozyme L) preparations. The conversion of TMP-AcGGM using *Tr*Man5A or Viscozyme L was determined to be 28.7% ± 1.1 and 32.5% ± 1.2, respectively. Furthermore, like the incubations analyzed for the release of reducing sugars (Figure 2), the rate of conversion estimated from the first data point (8 h) was highest for the purest AcGGM preparation (AD) which also gave the highest end-point conversion, while the rate, as well as the degree of conversion, was lowest for the most impure preparation (UFR) (Figure 4). Notably, ~70–80% of the end-point final hydrolysis was achieved within 8 h incubation using *Tr*Man5A (Figure 4a), while with Viscozyme L the hydrolysis was slower, reaching ~35–48% of the endpoint at 8 h but with more than 85% of the achieved end-point hydrolysis reached after 24 h (Figure 4b).

It is an interesting observation that the rate and the total hydrolysis of the SSL-AcGGM preparations decreased with increased content of lignin (Figure 4, Table 1). Several factors may have influenced the enzymatic bioconversion. Enzyme inhibition by lignin is a plausible contributing factor. Notably, lignin and lignin-derived compounds from wood processing have been shown to inhibit cellulases [28,30]. Remarkably, the hydrolysis of UFR preparation with *Tr*Man5A or Viscozyme L resulted in ~two-fold lower conversion of AcGGM compared to AD preparation after 48 h (Figure 4). A likely explanation for this is the ~72- fold higher lignin content in the UFR preparation compared to the AD preparation, while the AcGGM monosugar composition, acetylation, and apparent Mw remained similar in the different preparations (Table 1, Figure S1).

Interestingly, an approximately two-fold higher conversion of AcGGM in the AD preparation was achieved compared to the AcGGM from thermomechanical pulping after 48 h of incubation with both *Tr*Man5A and Viscozyme L (Figure 4, Tables S2 and S3), despite that the lignin content in the AD preparation was ~two-fold higher than for TMP-AcGGM. Also, the apparent Mw appeared to be similar for the AD preparation (Figure 1) and TMP-AcGGM [41]. The lower conversion with TMP-AcGGM was likely a result of the ~two-fold higher degree of acetylation of the mannan backbone in TMP-AcGGM [41] compared to the AcGGM in the AD preparation (Section 2.1.1). The effect of acetylation on reduced enzymatic hydrolysis of biomass from woods, in general, has been reviewed by Pawar et al. [53], and has specifically been reported for β-mannanases, including *Tr*Man5A [54,55]. In addition, thermal treatment of lignocellulose may generate lignin-aggregates that bind and inhibit enzymes such as cellulases [29,35,56]. However, if this occurred in the present case, it was probably a less pronounced contributing effect with the TMP-AcGGM, since it was purified with SEC [40], which likely separated such aggregates.

### 2.4. Hydrolysis of Acetyl-Galactoglucomannan at Different Enzyme Loadings

**Figure 5:**
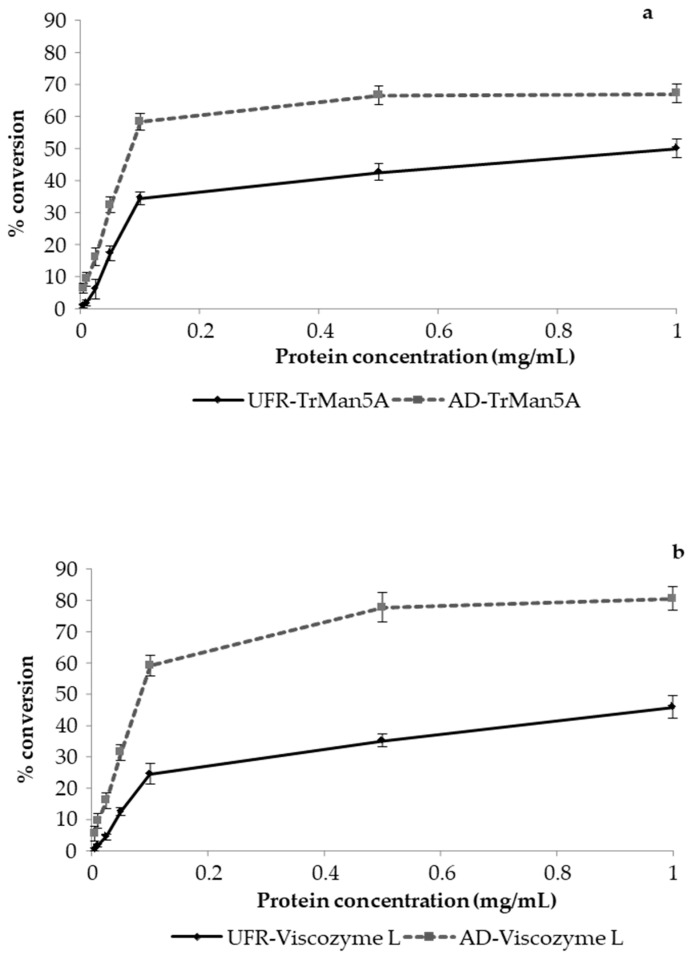
Hydrolysis of UFR and AD preparations using different concentrations of (**a**) *Tr*Man5A (**b**) Viscozyme L. *Tr*Man5A or Viscozyme L were used at seven different concentrations (0.005, 0.01, 0.025, 0.05, 0.1, 0.5 and 1.0 mg/mL) for hydrolysis of the SSL-AcGGM preparations UFR and AD. The substrates were used at an initial concentration of 100 mg/mL of GGM and the enzymatic hydrolysis was carried out over 24 h of incubation at 40 °C and pH 5.0. The reducing sugar equivalents were determined using the DNS method and the % conversion was calculated using a mannose standard curve.

To further investigate the effect of lignin on enzymatic bioconversion, the UFR and AD preparations with the highest and lowest lignin content, respectively, were hydrolyzed with *Tr*Man5A or Viscozyme L at different protein (i.e., enzyme) concentrations (0.005–1.0 mg/mL) for 24 h and the degree of conversion (%) was calculated based on reducing sugar equivalents (Figure 5). Regarding *Tr*Man5A and Viscozyme L, a significant increase in hydrolysis was observed with an increase in protein loading from 0.005 to 0.1 mg/L for the AD fraction, while the increase in hydrolysis for the UFR fraction was significantly lower compared to the AD fraction. (Figure 5a,b). Notably, with *Tr*Man5A or Viscozyme L, the maximum hydrolysis achieved with AD preparations was ~1.3 and ~1.8 folds higher, respectively, compared to the UFR preparation. Furthermore, similar bioconversion of AcGGM in the UFR preparation using *Tr*Man5A (49.9% ± 3.0) and Viscozyme L (45.9% ± 3.6) was observed at the highest protein loading (1.0 mg/mL). The ~73-fold higher total lignin (Table 1) content in the UFR fraction indicates the negative effect of lignin on enzymatic hydrolysis of the AcGGM preparations, although the presence of other inhibitors cannot be ruled out.

Lignin may be altered during wood processing [57]. The lignin in the current SSL (and thus the AcGGM preparations) was partially sulfonated (11% by weight) [25,26]. Increased hydrophilicity due to lignin sulfonation may decrease unproductive lignin-binding of cellulases when acting on the insoluble substrate [57,58,59]. However, Zheng et al. [32] observed a ~25% reduction in Avicel cellulose-hydrolysis by cellulase when 1% (*w*/*v*) lignosulfonate was added. Nonetheless, current understanding regarding the possible inhibitory effect of lignosulfonates on β-mannanases is lacking, and only a few studies with cellulases have been documented [32,60]. Lignosulfonates are amphiphilic in nature and have both hydrophilic and hydrophobic groups [61]. It has been discussed that the hydrophobic region of lignosulfonate could potentially bind to both Avicel and cellulase to form complexes that reduce enzyme-substrate interaction and thus hydrolysis [32]. Extrapolating this to our study, hydrophobic interactions between lignosulfonate and a carbohydrate-binding module (CBM) [62] carried by *Tr*Man5A and/or with hydrophobic residues in the active site of galactoglucomannan hydrolyzing enzymes [63] may have taken place. This may be one of the factors that could explain the reduced hydrolytic efficiency of both *Tr*Man5A and Viscozyme L during hydrolysis of the UFR fraction compared to the AD fraction.

Another factor that could potentially influence the low enzymatic conversion observed with the UFR fraction is the presence of water-soluble low molecular weight lignin (LMWL) compounds. In this study, the membrane filtration with an RC700PP membrane (10 kDa cut-off) is expected to remove most of the LMWL in the UFR fractions; however, membrane fouling and film formation could lead to retention of LMWL in the fractions [15,25]. The inhibitory effect of some LMWL compounds on certain mannanolytic enzymes has been studied [33,35,59].

In addition to lignosulfonates and LMWL, the potential role of lignin carbohydrate complexes (LCC) in terms of negatively influencing the hydrolytic efficiency of *Tr*Man5A and Viscozyme L also needs to be discussed. In this study, SEC analysis of all the fractions showed overlapping regions of both RI and UV signals (Figure 1 and Figure S1), indicating the possible presence of LCC, which, in low abundancy, has been indicated previously in SSL-AcGGM, also with more specific methods [16]. The role of LCC in steric hindrance and non-specific binding of enzymes such as cellulases has been evaluated [64,65,66]. The fact that even with a ten-fold higher enzyme load, the conversion of UFR (at 1 mg/mL enzyme) was 20–30% lower than for AD (at 0.1 mg/mL enzyme) (Figure 4) may be a sign that the presence of lignin sterically hinders conversion, and that the effect is more pronounced when the more accessible AcGGM has been hydrolyzed. A possible hypothesis is that the presence of LCC facilitates interaction between unbound lignin and/or lignosulfonates and AcGGM, which could lead to a shielding effect. The view that part of the AcGGM is more accessible and that restricted AcGGM is enriched during hydrolysis is also strengthened by the conversion profiles in Figure 2 and by the observation that increasing the enzyme load beyond 0.1 mg/mL has a much more limited effect on the conversion than the increase from 0.005 to 0.1 mg/mL (Figure 5).

The inhibitory effect of insoluble lignin on cellulases and their mechanism of inhibition has been studied extensively [30,31,59,67,68]. However, the specific effect of lignin, lignosulfonates, and LCCs on β-mannan-converting enzymes, the mechanism(s) involved, as well how to overcome this hurdle in the conversion of crude hemicellulose preparations is an interesting future subject to study.

### 2.5. Acetyl-Glactoglucomannan Biorefining and Future Prospectives

To maximize the valorization potential of biomass, the production of high value biochemicals and saccharides is an attractive route. However, it is important to identify the product of interest and its purity relative to the requirements of a given application. Purification/pretreatment measures and often high enzyme dosage requirements are two main contributors to processing costs in enzymatic saccharification, as observed in studies involving lignocellulosic feedstocks [69,70]. For instance, in this study, we demonstrated an effective purification strategy that resulted in 87.3% pure GGM in the purest preparation (AD) due to the highly selective removal of lignin. We also illustrated that Viscozyme L is an efficient commercial cocktail for the hydrolysis of the AcGGM present in SSL, with more than 80% hydrolysis achieved with the purest fraction at high enzyme loadings (Figure 5). For total saccharification, it is likely that Viscozyme L incubations could be supplemented with additional enzyme activities/specificities. Therefore, custom-designed cocktails based on enzyme synergy studies taking into consideration the composition of the waste material could make a biorefining process cost-effective.

With an eye on potential valorization, the scope of the current study may be extended to several applications for enzymatically produced AcGGM-saccharides, including prebiotics [24], bioethanol production [71], and chemo-enzymatic synthesis of glyco-chemicals [22]. Not only saccharides, but lignosulfonates separated during the purification process might have industrial applications, including usage as plasticizers, flocculants, metal absorbents, composites, etc. [72].

## 3. Materials and Methods

### 3.1. Materials

Celluclast 1.5 L, Cellic CTec 2, Viscozyme L (manufactured by Novozymes Corp.), oat-spelt xylan (95590), Amberlite XAD4, and Amberlite IRA958 were purchased from Sigma (St. Louis, MO, USA). The *T. reesei* β-mannanase (*Tr*Man5A) was obtained as previously described by Hägglund et al. [73]. AcGGM from thermomechanical pulping (TMP-AcGGM) was obtained as described in Andersson et al. [40]. Mannobiose (M2), mannotriose (M3), mannotetraose (M4), mannopentaose (M5), mannohexaose (M6), galactosyl-mannotriose (GM3), di-galactosyl-mannopentaose (G2M5), para-nitrophenyl (*p*NP)-α-D-galactopyranoside, *p*NP-β-D-glucopyranoside, *p*NP-β-D-mannopyranoside, *p*NP-β-D-xylopyranoside and low viscosity locust bean gum (lvLBG) were purchased from Megazyme (Bray, Ireland). Acetone and pullulan molecular weight standards were procured from Merck (Darmstadt, Germany).

### 3.2. Purification of Acetyl Galactoglucomannan from Spent Sulfite Liquor (SSL-AcGGM)

#### 3.2.1. Membrane Filtration

The starting material used in the present study was derived from an ultrafiltration pilot plant situated on-site at the Domsjö Fabriker bio-refinery in Örnsköldsvik, Sweden, which uses softwood raw material [74]; this was termed SSL retentate. The feed for the pilot plant was SSL (62,500.0 g); the conditions used for the filtration and the composition of the untreated SSL have been published previously [26]. The SSL retentate was concentrated in a lab-scale membrane filtration unit.

The membrane filtration setup consisted of a 400 mL stirred cell with an RC70PP membrane (Alfa Laval Nordic A/S, Søborg, Denmark), and 50 mm magnetic rod placed on top of a heating plate with a built-in magnetic stirrer (MR2002, Heidolph Instruments GmbH & Co.KG, Schwabach, Germany) from which the cross-flow velocity was set [25]. The filtrations were performed in fed-batch mode with a 15 L feed tank from which the pressure for the system was also set using nitrogen gas connected to a gas valve. The pressure was monitored and transmitted to a personal computer using an electronic pressure gauge (DCS40.0AR, Trafag AG, Bubikon, Switzerland). The flux or permeate flow was monitored using a balance (PL6001-I, Mettler Toledo Inc., Columbus, OH, USA) also connected to a personal computer that ran Lab View (NI Co., Austin, TX, USA). The temperature, crossflow velocity, and transmembrane pressure were 50 °C, 0.5 m/s, and 5.5 bar respectively. The procedure was repeated for a total of 16 runs to produce enough concentrated retentate for the forthcoming purification steps. The retentate was concentrated in fed-batch mode to a total volume reduction of 90%. The final amount of the retentate was 6250 g after membrane filtration, out of which 250 g was separated for analysis and 6000 g was used for the next purification step. In this paper, the concentrated retentate was given the designation ultrafiltration retentate (UFR).

#### 3.2.2. Antisolvent Precipitation

Precipitation was performed using acetone (SupraSolv MS, Merck Schuchardt OHG, Hohenbrunn, Germany) as antisolvent at a final concentration of 50 wt. %, following a previous study [16]. The acetone was slowly added to 1 kg of UFR under agitation (using a shaker at 50 r.p.m.) at room temperature until the final weight of the mixture reached 2 kg. The acetone addition took approximately 15 min and the mixture was left to agitate for an additional 15 min. The mixture was then transferred to four 750 mL centrifuge bottles (Beckman Coulter, Brea, CA, USA) that were centrifuged at 4000 rpm for 20 min (Jouan S.A., Model C412, Saint-herblain, Nantes, France) after which the liquid was carefully decanted. The remaining solids were dried in an oven at 50 °C for 48 h to remove any remaining acetone in the precipitate. The dry solids were redissolved in deionized water to the same initial total weight of 1 kg before moving on to the next treatment step. This procedure was repeated until all the concentrated retentates were treated. The antisolvent precipitated preparation was given the designation antisolvent-precipitation concentrate (AC). Finally, 250 g of the AC concentrate was separated for analysis and 5750 g was used in the next purification step.

#### 3.2.3. Adsorption

**Figure 6:**
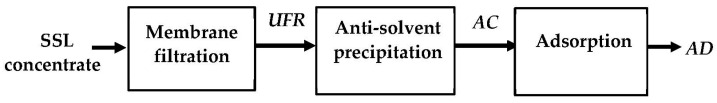
Schematics illustrating the purification scheme for purification of acetylated galactoglucomannan (AcGGM) from SSL concentrate. SSL concentrate is the starting material generated after the SSL was concentrated onsite at the softwood biorefinery using an ultrafiltration device. UFR: ultrafiltration retentate; AC: antisolvent-precipitation concentrate; AD: adsorption preparation.

Two XK 50 columns, (50 mm diameter; 250 mm length) (GE Healthcare Bio-Sciences AB, Uppsala, Sweden) were used of which the first column was loaded with 350 g of Amberlite XAD4 and the second with 400 g of Amberlite IRA958 (Sigma-Aldrich, Saint Louis, MO, USA) [27]. The flow rate in these columns was 1 mL/min (GP 50 gradient pump, Thermo Fisher Scientific Inc., Waltham, MA, USA) for both the adsorption and wash sequence. After a solution had been pumped through a column, the column was emptied by purging with air using a hose pump. XAD4 was cleaned with 700 mL of 10% ethanol solution and subsequently washed with 1400 mL of deionized water. 5750 g of the AC solution was passed through the XAD4 column after which a sample of 250 g was removed from the adsorption column permeate. The second column (IRA958) was washed with 1400 mL of deionized water and 5500 g of permeate was passed through this column, producing the purest permeate in this series, which was designated adsorption preparation (AD). The yield for each purification step was calculated based on the galactoglucomannan content (g) before and after each purification step and was expressed as % yield. The purification scheme is illustrated in Figure 6.

### 3.3. Characterization of the Material

#### 3.3.1. Analysis of Acid-Insoluble Solids and Total Carbohydrate Content

The amount of polymeric and oligomeric hemicellulose in the samples was measured by acid-hydrolyzing 10 mL of the samples with 0.75 mL of 72% sulfuric acid at 121 °C for 1 h in an autoclave (Systec DX 150, Wittenberg, Germany) [39]. The samples were filtered after the hydrolysis to remove the acid-insoluble solids and the filters were dried at 105 °C for 24 h. The acid-insoluble solids content was gravimetrically determined by weighing the filters after the drying process. The remaining liquid was analyzed with high-performance anion-exchange chromatography with pulsed amperometric detection (HPAEC-PAD) (ICS-5000+, Thermo Fisher Scientific Inc., Waltham, MA, USA) for the monomeric sugar content. The column used for the separation was a CarboPac PA1 analytical column (Thermo Fisher Scientific Inc., Waltham, MA, USA) with deionized water as eluent at a 1 mL/min flow rate with 200 mM sodium hydroxide post-column addition at a flow rate of 0.5 mL/min. The calibration standards used were L-arabinose, D-galactose, D-glucose, D-xylose, and D-mannose all of which were supplied by Fluka Chemie AG (Buchs, Switzerland). The monosugar content was anhydro corrected (0.88 for pentoses and 0.90 for hexoses) to determine the hemicellulose content in the solution [75]. The amount of free monosaccharides in the different preparations was determined by using the same HPAEC-PAD method without the acid-hydrolysis step and anhydro corrections.

#### 3.3.2. Estimation of Lignin Content

The lignin content including the lignosulfonate in the solutions was determined using a UV spectrophotometer (Shimadzu UV-1800, Kyoto, Japan) at 280 nm wavelength and an extinction coefficient of 13.01 L/g cm [25]. The samples were diluted using deionized water before the UV measurements.

#### 3.3.3. Lyophilization and Resuspension of AcGGM Preparations

The three different preparations (UFR, AC, and AD) obtained during the purification of SSL-AcGGM were water-soluble. They had the following gravimetrical dry substances (mg/g): 239.86 (UFR), 89.79 (AC), and 61.83 (AD). Aliquots from each were lyophilized and suspended in 100 mM acetate buffer (pH 5.0). Stock solutions were prepared and a concentration of 100 mg/mL of galactoglucomannan (GGM) was obtained in the enzymatic conversion reaction mixtures, based on the three polymeric monosugar units in AcGGM (i.e., galactan glucan, mannan).

#### 3.3.4. Acetyl Content Determination

The acetyl content in the AcGGM from UFR, AC, and AD preparations was determined by de-acetylation of 35 mg/mL of AcGGM using 12.5% ammonia solution as described by Jacobs et al. [76]. The acetic acid content in the AcGGM before and after de-acetylation was analyzed with a variable wavelength UV detector at 280 nm using high-performance liquid chromatography (HPLC) (Jasco LC-4000 series, Jasco, Easton, MD, USA), with an Aminex HPX-87H column (Bio-Rad, Hercules, CA, USA) and eluting with 0.5 mM sulfuric acid at 40 °C [77]. Acetic acid was used as standard. The control value (free acetic acid in the sample) was subtracted, and the acetyl content was represented as the weight of the acetyl in mg/g of the original SSL-AcGGM preparations. The samples were analyzed in triplicate and mean values ± SD (standard deviation) are presented.

#### 3.3.5. Size Exclusion Chromatography

The molecular weight (Mw) distribution was determined using size exclusion chromatography (SEC). UFR, AC, and AD preparations were loaded at 1 mg/mL of AcGGM on a Dionex Ultimate 3000 HPLC system (Thermo Fisher, USA). For aqueous lignocellulose extracts rich in carbohydrates, lignin, and lignin-derivates, refractive index (RI) can be used for general detection and UV for detection of lignin and its derivates [12,26]. Pullulan [78] or dextran [12] has previously been used as SEC standards for AcGGM. The analytical SEC separation was achieved by injecting 10.0 µL of the analytes into a Shodex, SB-803-HQ column (Shodex, Tokyo, Japan) with a variable wavelength UV detector at 280 nm (Thermo Fisher, USA) and an RI 101 detector (Shodex, Tokyo, Japan). Water was used as an eluent at a flow rate of 0.5 mL/min and the temperature was maintained at 25 °C. Pullulan molecular weight standards (0.3, 1.6, 6.3, 9.3, 22.8, 50.6, and 116.0 kDa) were used. A logarithmic (log Mw vs. retention time) plot was used for the estimation of apparent Mw. The samples were analyzed in triplicate.

### 3.4. Enzymatic Conversion

#### 3.4.1. Hydrolytic Efficiency of Commercial Enzyme Cocktails and TrMan5A

The commercial cocktails Celluclast 1.5 L, Cellic CTec 2, and Viscozyme L, and the β-mannanase *Tr*Man5A from *T. reesei* [73] were used to evaluate the hydrolysis of AcGGM using the well-characterized TMP-AcGGM (41). The TMP-AcGGM has 77.0% AcGGM content in a molar ratio of mannosyl:glucosyl:galactosyl:acetyl as 1:0.3:0.3:0.4. The hydrolysis was carried out at 40 °C, pH 5.0 (100 mM acetate buffer) for 24 h, 100 rpm using 100 g/L of GGM. The commercial enzyme cocktails and *Tr*Man5A were used at a 0.1 mg/mL protein concentration. The protein content was determined using Bradford Protein Assay Kit with bovine serum albumin as the standard (Bio-Rad, Hercules, CA, USA). The reducing sugar content was analyzed using the 3,5-dinitrosalicylic acid (DNS) method [79] using a mannose standard curve and was reported as % conversion (reducing sugar equivalents). The reactions were carried out in triplicate and mean values ± SD are presented. Based on the degree of conversion (%), Viscozyme L and *Tr*Man5A were selected for further studies.

#### 3.4.2. Enzyme Activity and Stability

The hydrolytic activity of the commercial enzyme cocktails and *Tr*Man5A was determined by separately incubating 0.1 mg/mL (protein concentration) of either preparation with different substrates. The β-mannanase activity was determined with 0.5% low viscosity locust bean gum (lvLBG) (Megazyme) as the substrate at pH 5.0 (50 mM acetate buffer) by DNS assay to detect the produced reducing sugars [42,79]. Xylanase activity was determined similarly, but with 1% beechwood xylan (Megazyme) as the substrate in the same acetate buffer [42]. α-Galactosidase, β-glucosidase, β-xylosidase, and β-mannosidase activities were determined based on *p*-nitrophenol release from 1 mM *p*NP-α-D-galactopyranoside, *p*NP-β-D-glucopyranoside, *p*NP-D-xylopyranoside, and *p*NP-β-D-mannopyranoside, respectively, using the same acetate buffer [42]. The stability of Viscozyme L and *Tr*Man5A at 0.1 mg/mL protein concentration was studied by incubating the enzyme (s) at 40 °C and pH 5.0 (100 mM acetate buffer) over 48 h and analyzing the residual enzyme activities at different time points (0, 4, 8, 24 and 48 h). In the enzyme stability measurements, the detected enzyme activities were normalized to those at 0 h and reported as % residual activity. The reactions were carried out in triplicate and mean values ± SD are presented.

#### 3.4.3. Effect of Purification on Hydrolysis of Acetyl-Galactoglucomannan

The UFR, AC, and AD SSL-AcGGM preparations obtained after the different purification steps, as well as TMP-AcGGM, were hydrolyzed using Viscozyme L (0.1 mg/mL) and *Tr*Man5A (0.1 mg/mL) at 40 °C for 48 h, 100 rpm, and pH 5.0. GGM was used at a concentration of 100 g/L in the reaction mixture. Sampling was carried out at 0, 4, 8, 12, 24, and 48 h. The reducing sugars were quantified using the DNS method. The reactions were carried out in triplicate. The monosaccharide and the oligosaccharide analysis for 0, 8, 24, and 48 h samples (filtered with 0.22 um syringe filter) were carried out using the HPAEC-PAD (ICS-5000, Thermo Fisher Scientific Inc., Waltham, MA, USA) [24]. All the samples were filtered (0.22 um, syringe filter) and were subjected to monosaccharide and oligosaccharide analysis. For monosaccharide analysis, the separation was achieved using a CarboPac PA20 column (150 mm × 3, 6.5 µm) and a guard column (30 mm × 3 mm) of the same material. The elution was carried out using an isocratic flow of 0.45 mM NaOH for 22 min followed by washing with 100 mM NaOH for 5 min and re-equilibration with 0.45 mM NaOH for 5 min [24]. The flow rate was maintained at 0.5 mL/min. D-Galactose, D-glucose, and D-mannose were used as standards. A CarboPac PA200 column (250 mm × 3, 5.5 μm) and a guard column (231 mm × 3 mm) of the same material were used for oligosaccharide analysis. The elution was carried out with an isocratic flow of 17 mM NaOH for 20 min followed by a 17 mM to 80 mM NaOH gradient for 20 min, washing with 200 mM NaOH + 100 mM NaOAc for 5 min, and re-equilibration with 17 mM NaOH for 10 min [24]. The flow rate was 0.4 mL/min. The samples were analyzed in triplicate. Commercially available mannan-oligosaccharide (MOS), M2, M3, M4, M5, M6, GM3, and G2M5 were used as standards. In cases when a product was eluted at the same time as one of the known standards, the product concentration was determined using an external calibration curve. The quantities of quantifiable monosaccharides and oligosaccharides after enzymatic hydrolysis were reported as % conversion (sum of produced oligosaccharides as monomer equivalents and mono-saccharides/total galactan, glucan, and mannan content of the original substrate in monomer equivalents). Mean values ± SD are presented.

#### 3.4.4. Effect of Protein Loading on Enzymatic Hydrolysis of Acetyl-Galactoglucomannan

The effect of protein (enzyme) loading on AcGGM hydrolysis was evaluated by varying the protein loading of Viscozyme L or *Tr*Man5A over a range of 0.005 to 1.0 mg/mL. The UFR and AD preparations of the SSL-AcGGM were used at a substrate concentration of 100 mg/mL. Hydrolysis was carried out for 24 h at 40 °C and pH 5.0. The reactions were carried out in triplicate. The reducing sugar content and percentage conversion were measured with the DNS method, as described in Section 3.4.1. Mean values ± SD are presented.

## 4. Conclusions

In the present study, we demonstrated an efficient purification procedure for the successful removal of lignin from a refined softwood-derived side stream (SSL), with low loss of AcGGM polysaccharides. The commercial enzyme cocktail Viscozyme L was efficient in the saccharification of AcGGM preparations with a higher degree of conversion (%) compared with a well-established mono-component β-mannanase, *Tr*Man5A from *T. reesei*. This highlights that a combination of enzymes, in this case, α-galactosidase, β-glucosidase, and β-mannosidase (Table S1), in addition to backbone cleaving β-mannanase, is required for the efficient conversion of AcGGM. Furthermore, the study highlighted how purification significantly improved the bioconversion of AcGGM using both Viscozyme L and *Tr*Man5A, and that lignin and/or lignosulfonates are plausible components for the reduction of enzymatic conversion. The mechanism(s) involved need to be further investigated, but enzyme inhibition, as well as steric hindrance, are suggestions. Thus, this study envisages the valorization of AcGGM, which is abundantly present in softwood-based side streams, using Viscozyme L and/or *Tr*Man5A, and emphasizes the need for further studies to understand the effect of substrate purity and the inhibitory role of soluble lignin on enzymes which convert AcGGM and other β-mannans.

## Figures and Tables

**Table 1:** Composition of SSL after different purification steps.

All Values in (mg/g)	UFR	AC	AD
Ash	36.81	11.56	2.56
Total lignin	192.16	25.44	2.65
Klason lignin	5.27	0.88	1.57
Arabinan	0.75	0.41	0.35
Galactan	9.62	8.92	8.04
Glucan	8.75	7.43	6.69
Xylan	2.89	2.45	2.19
Mannan	25.52	23.59	21.56
Arabinose	0.32	0.046	0.034
Galactose	0.09	0.015	0.002
Glucose	0.12	0.011	0.003
Xylose	0.11	0.003	0.019
Mannose	0.11	0.017	0.011

UFR: preparation obtained after membrane filtration of retentate obtained from on-site ultrafiltration unit; AC: acetone precipitated preparation; AD: adsorption preparation. All the preparations were obtained during consecutive purification of SSL as mentioned under Section 3.2. The carbohydrate content was determined by high-performance anion-exchange chromatography with pulsed amperometric detection (HPAEC-PAD) using CarboPac PA1 analytical column. The standard deviations were < 5% in all cases. Lignin content was determined by measuring the absorbance at 280 nm. The purity of the GGM in the preparations was determined (% of dry weight with total lignin but excluding ash) to be 18.3%, 58.5%, and 87.3% for UFR, AC, and AD preparations, respectively.

## Data Availability

The data presented in this study are available in Supplementary Materials.

## Supplementary Materials

The following supporting information can be downloaded at: https://www.mdpi.com/article/10.3390/molecules27103207/s1, Figure S1: Size exclusion chromatography of different preparation from SSL-AcGGM, (a) RI, (b) UV, detector response; Figure S2: Hydrolysis of TMP-AcGGM using different commercial enzyme cocktails and purified β-mannanase from *T. reesei* (*Tr*Man5A); Figure S3: Stability of Viscozyme L and *Tr*Man5A at 40 °C and pH 5.0 over 48 h; Figure S4: Product profile based on HPAEC-PAD analysis of hydrolysates obtained after hydrolysis of AcGGM from different SSL fractions and TMP-AcGGM over 8 and 48 h using *Tr*Man5A; Figure S5: Product profile based on HPAEC-PAD analysis of hydrolysates obtained after hydrolysis of AcGGM from different SSL fractions and TMP-AcGGM over 8 and 48 h using Viscozyme L. Table S1: Enzyme activities detected in different commercial cocktails; Table S2: Major products quantified after 8 and 48 h hydrolysis of AcGGM from different SSL preparations and TMP using *Tr*Man5A; Table S3: Major products quantified after 8 and 48 h hydrolysis of AcGGM from different SSL preparations and TMP using Viscozyme L.
