# Supplementary material for: Enzymatic Conversion of Different Qualities of Refined Softwood Hemicellulose Recovered from Spent Sulfite Liquor
Source: Molecules. 2022 May 17;27(10):3207. doi: 10.3390/molecules27103207 (PMC9143570; doi:10.3390/molecules27103207)
Supplement: Supplementary file 1 [file molecules-27-03207-s001.zip › molecules-1693756-supplementary.pdf]

Supplementary Material

For **Enzymatic Conversion of Different Qualities of Refined Softwood Hemicellulose Recovered from Spent Sulfite Liquor**

By **Abhishek Bhattacharya, Samuel Butler, Basel Al-Rudainy, Ola Wallberg and Henrik Stålbrand** (Lund University)

Contact: Henrik.stalbrand@biochemistry.lu.se; Journal: Molecules (published by mdpi)

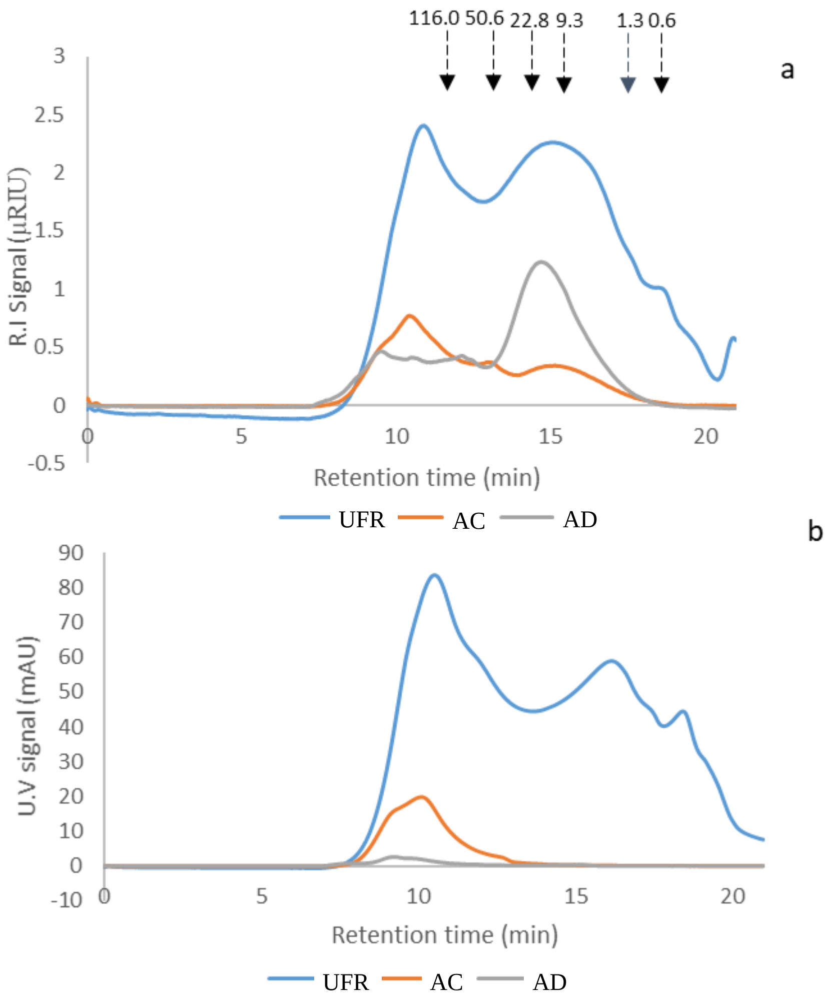

Figure. S1. Size exclusion chromatography of different preparation from SSL-AcGGM, (**a**) RI, (**b**) UV, detector response.
The UFR, AC and AD preparations were analysed at 1g/L of GGM using water as eluent based on RI (µRIU) and UV (mAU) response. Arrows (dashed) point to the peak maximum of various pullulan standards (kDa).

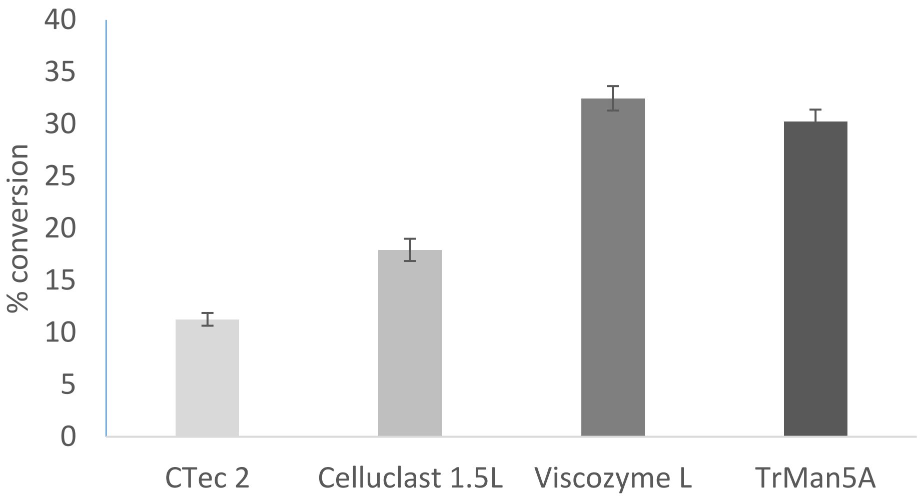

Figure S2. Hydrolysis of TMP-AcGGM using different commercial enzyme cocktails and purified β-mannanase from *T. reesei* (*Tr*Man5A).

The substrate was used at an initial concentration of 100 mg/mL of GGM. The commercial enzyme cocktails and *Tr*Man5A were used at 0.1 mg/mL protein loadings. The hydrolysis reactions were carried out at 40 ^{o}C, pH 5.0 for 24 h. The reducing sugar content was analysed using DNS method with a mannose standard curve and was reported as % conversion.

Table S1. Enzyme activities detected in different commercial cocktails.

**Cocktail/Enzyme**	**Mannanase (U/ml)**	**Xylanase (U/ml)**	**α-Gal (U/ml)**	**β-Man (U/ml)**	**β-Glu (U/ml)**	**β-Xly (U/ml)**
**CTec 2**	N.D	3610 ± 69.0	76.1 ± 6	42.8 ± 0.5	576.3 ± 48	N.M
**Celluclast 1.5L**	417.4 ± 26	316.4 ± 32	34.5 ± 4	N.M	288.5 ± 19	N.M
**Viscozyme L**	4191.7 ± 97	192.8 ± 12	364.1 ± 31	29.6 ± 0.3	169.7 ± 17	N.D

Enzyme activity reported as U/ml; U=μmoles of product formed per min. α-Gal: α-galactosidase activity, β-Man: β-mannosidase activity, β-Glu: β-glucosidase activity, β-Xyl: β- xylosidase activity. Mannose was used as standard curve for determination of reducing sugar equivalents with low viscosity locust bean gum (0.5%) and beechwood xylan (1.0 %) as substrates for determination of β-mannanase and β-xylanase activity, respectively. p-Nitrophenol was used for a standard curve for determination of p-nitrophenol release from *p*NP -α-galactopyranoside, *p*NP -β-mannopyranoside, *p*NP -β-glucopyranoside and *p*NP -β-xylopyranoside for determination of α-galactosidase, β-mannosidase, β-glucosidase and β- xylosidase activity, respectively. All the *p*NP substrates were used at 1mM concentration. The commercial enzyme cocktails and *Tr*Man5A were used at 0.1 mg/mL protein loadings. N.D-not detected, N.M-not measured.

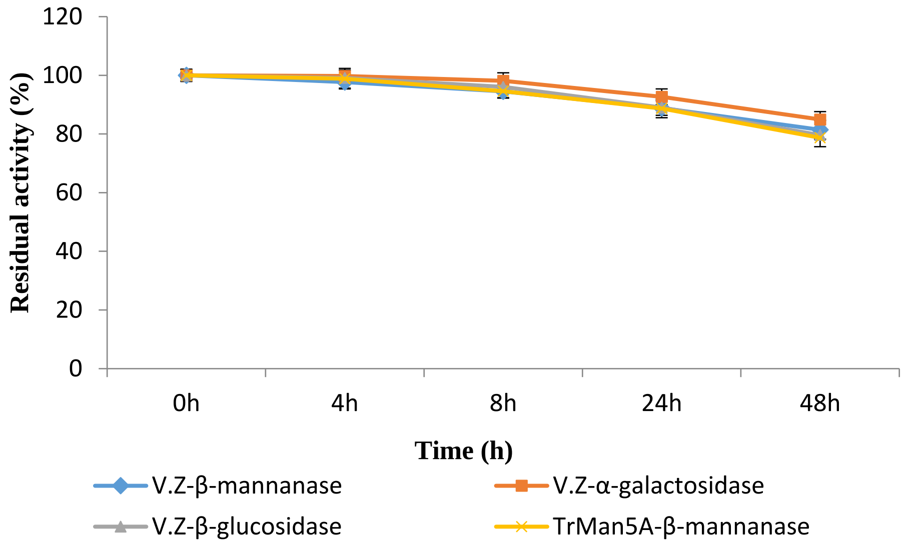

Figure S3. Stability of Viscozyme L and TrMan5A at 40°C and pH 5.0 over 48 h. The stability of β-mannanase, α-galactosidase and β-glucosidase activities in Viscozyme L (V.Z) and β-mannanase activity of *Tr*Man5A was determined by incubating Viscozyme L or *Tr*Man5A at protein concentration of 0.1 mg/mL with 100 mM acetate buffer, pH 5.0 at 40 ^{o}C over 48 h and determining the residual activity at periodic intervals. At 0 h, the β-mannanase, α-galactosidase and β-glucosidase activities in Viscozyme L were determined to be 293.5 µmoles/min, 24.3 µmoles/min and 11.3 µmoles/min, respectively and considered as 100%, while *Tr*Man5A, β-mannanase activity at 0 h was determined to be 230.4 µmoles/min and was considered as 100%. Low viscosity locust bean gum (0.5%) and *p*NP -β-mannopyranoside,, *p*NP -α-galactopyranoside, *p*NP -β-glucopyranoside each at 1mM concentration were used to determine the β-mannanase, α-galactosidase and β-glucosidase activity, respectively.

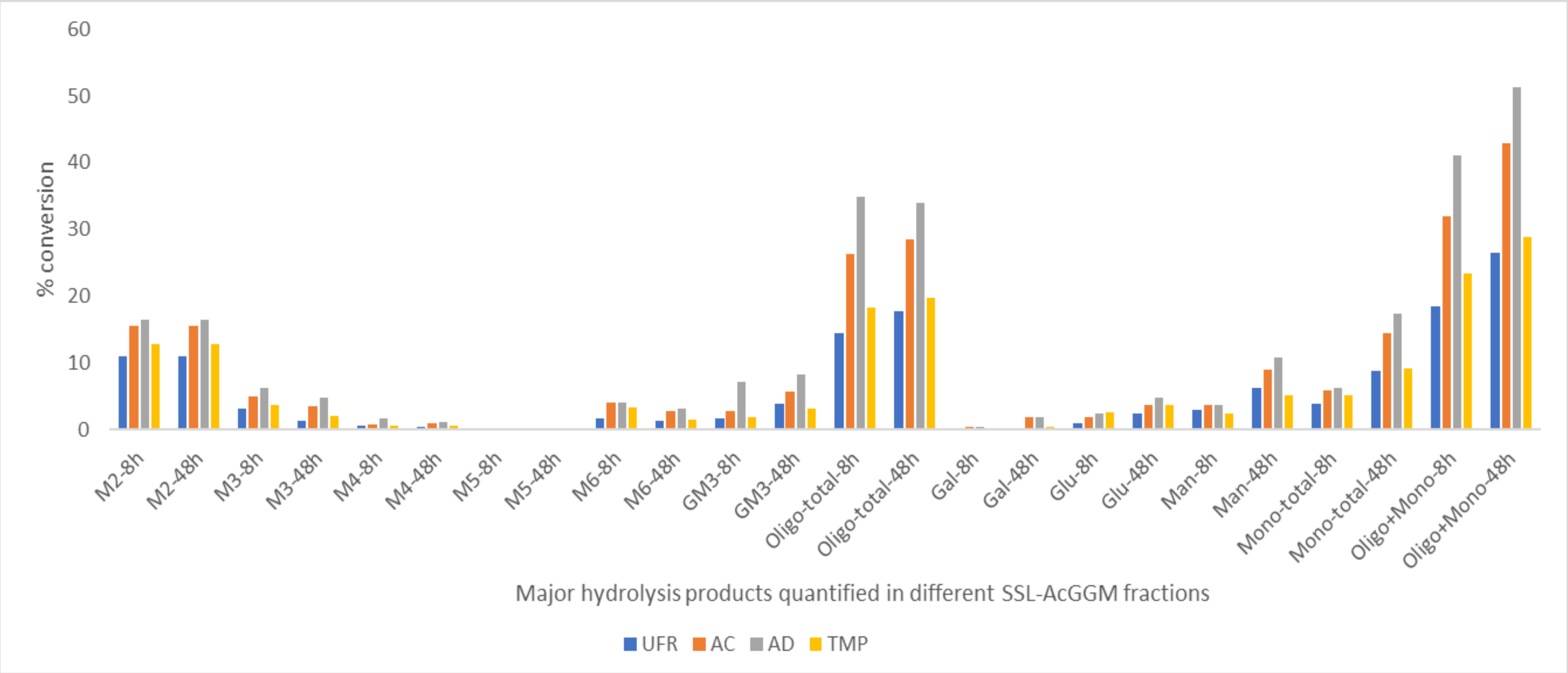

Figure S4. Product profile based on HPAEC-PAD analysis of hydrolysates obtained after hydrolysis of AcGGM from different SSL fractions and TMP-AcGGM over 8 and 48h using *Tr*Man5A. M2, M3, M4, M5, M6, and GM3 correspond to mannobiose, mannotriose, mannotetraose, mannopentaose, mannohexaose, and galactosyl mannotriose, respectively. Mono is for monosaccharides and oligo is for oligosaccharides. 8h and 48h correspond to the incubation time of different AcGGM samples with *Tr*Man5A. Oligosaccharides and monosaccharides were quantified based on the products eluting with known standards with HPAEC-PAD using CarboPac PA200 and PA20 columns, respectively. All substrates were used at 100 mg/mL of GGM. The quantification of mannan-oligosaccharides (in terms of monomer equivalents) and monosaccharides were compared to the monomer equivalents of polymeric mannan, galactan, and glucan, and the degree of conversion was reported as % conversion. The standard deviations were < 10 % in all cases. Numeric values are given in **Table S2**.

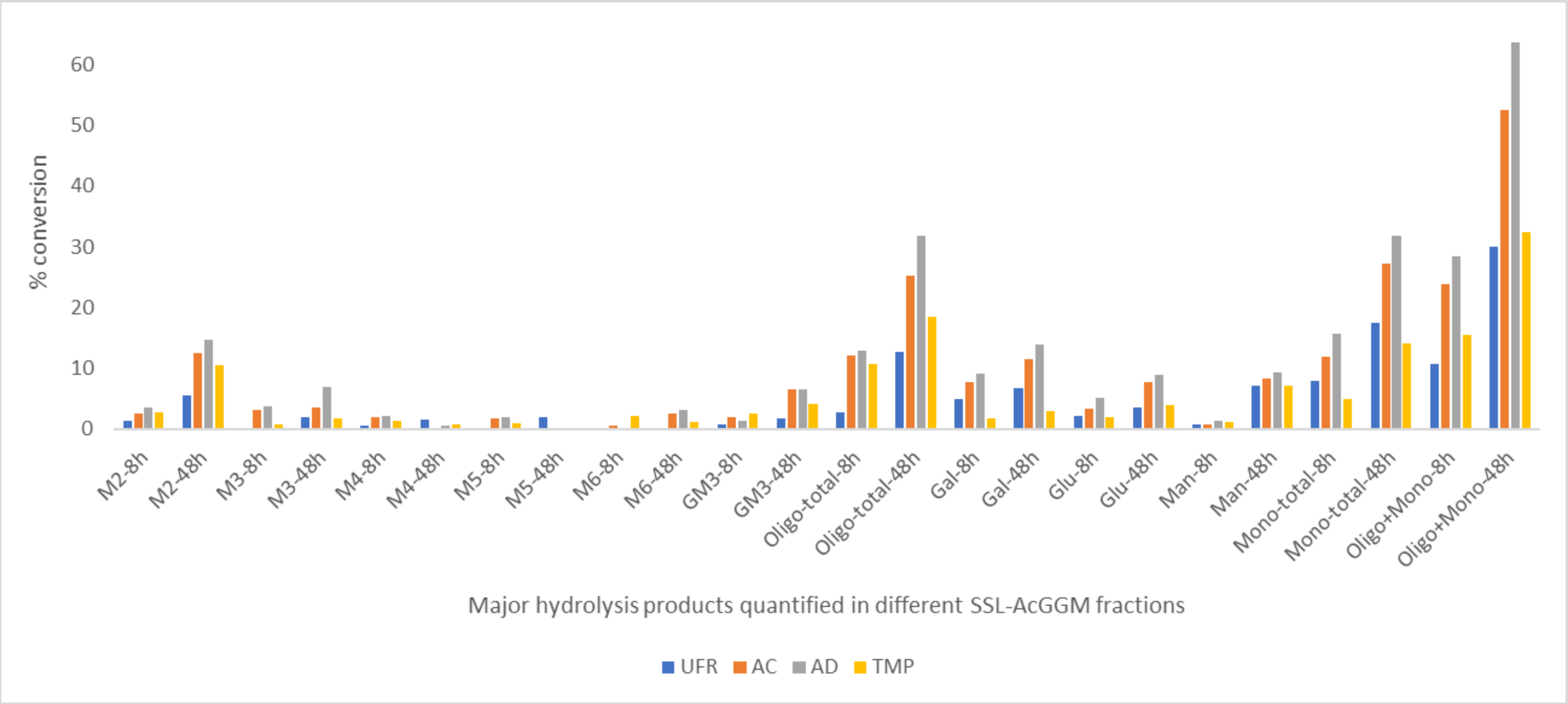

Figure S5. Product profile based on HPAEC-PAD analysis of hydrolysates obtained after hydrolysis of AcGGM from different SSL fractions and TMP-AcGGM over 8 and 48h using Viscozyme L. M2, M3, M4, M5, M6, and GM3 correspond to mannobiose, mannotriose, mannotetraose, mannopentaose, mannohexaose, and galactosyl mannotriose, respectively. Mono is for monosaccharides and oligo is for oligosaccharides. 8h and 48h correspond to the incubation time of different AcGGM samples with TrMan5A. Oligosaccharides and monosaccharides were quantified based on the products eluting with known standards with HPAEC-PAD using CarboPac PA200 and PA20 columns, respectively. All substrates were used at 100 mg/mL of GGM. The quantification of mannan-oligosaccharides (in terms of monomer equivalents) and monosaccharides were compared to the monomer equivalents of polymeric mannan, galactan, and glucan, and the degree of conversion was reported as % conversion. The standard deviations were < 10 % in all cases. Numeric values are given in **Table S3**.

Table S2. Major products quantified after 8 and 48h hydrolysis of AcGGM from different SSL preparations and TMP using *Tr*Man5A.

**Products**	**Oligosaccharide quantification (% conversion)**														**Monosaccharide quantification (% conversion)**							
	**M2**		**M3**		**M4**		**M5**		**M6**		**GM3**		**Oligos-total**		**Galactose**		**Glucose**		**Mannose**		**Mono-total**	
**AcGGM**	8h	48h	8h	48h	8h	48h	8h	48h	8h	48h	8h	48h	8h	48h	8h	48h	8h	48h	8h	48h	8h	48h
**UFR**	7.6	10.9	3.1	1.3	0.5	0.4	n.d	n.d	1.6	1.3	1.7	3.8	14.5	17.7	0.1	0.2	0.9	2.4	2.9	6.2	3.9	8.8
**AC**	13.7	15.6	4.9	3.4	0.8	1.0	n.d	n.d	4.0	2.7	2.8	5.6	26.2	28.4	0.3	1.8	1.9	3.7	3.6	8.9	5.8	14.5
**AD**	15.9	16.5	6.2	4.7	1.6	1.2	n.d	n.d	4.0	3.2	7.1	8.3	34.8	33.9	0.3	1.9	2.3	4.7	3.6	10.8	6.2	17.4
**TMP**	9.1	12.7	3.6	2.0	0.5	0.5	n.d	n.d	3.3	1.4	1.8	3.1	18.3	19.7	0.1	0.3	2.6	3.6	2.4	5.1	5.1	9.1

Oligosaccharides and monosaccharides were quantified based on known standards with HPAEC-PAD using CarboPac PA200 and PA20 columns, respectively. All substrates were used at 100 mg/mL of GGM. The quantification of mannan-oligosaccharides (in terms of monomer equivalents) and monosaccharides were compared to the monomer equivalents of polymeric mannan, galactan and glucan and the degree of conversion was reported as % conversion. The standard deviations were < 10 % in all cases. The same data as Figure S4.

Table S3. Major products quantified after 8 and 48h hydrolysis of AcGGM from different SSL preparations and TMP using Viscozyme L.

Products	Oligosaccharide quantification (% conversion)														Monosaccharide quantification (% conversion)							
	**M2**		**M3**		**M4**		**M5**		**M6**		**GM3**		**Oligos-total**		**Galactose**		**Glucose**		**Mannose**		**Mono-total**	
**AcGGM**	8h	48h	8h	48h	8h	48h	8h	48h	8h	48h	8h	48h	8h	48h	8h	48h	8h	48h	8h	48h	8h	48h
**UFR**	1.3	5.5	0.2	1.9	0.6	1.5	n.d	2.0	n.d	n.d	0.7	1.7	2.8	12.6	4.9	6.8	2.2	3.6	0.8	7.1	7.9	17.5
**AC**	2.6	12.5	3.1	3.6	2.0	0.2	1.8	n.d	0.5	2.5	2.0	6.5	12.0	25.3	7.7	11.4	3.4	7.6	0.8	8.2	11.9	27.2
**AD**	3.5	14.7	3.8	6.9	2.2	0.5	1.9	n.d	n.d	3.2	1.4	6.6	12.8	31.9	9.1	13.8	5.1	8.8	1.4	9.2	15.6	31.8
**TMP**	2.7	10.4	0.8	1.8	1.4	0.7	1.0	0.2	2.2	1.1	2.5	4.2	10.6	18.4	1.8	2.9	1.9	4.0	1.2	7.1	4.9	14.0

Oligosaccharides and monosaccharides were quantified based on known standards with HPAEC-PAD using CarboPac PA200 and PA20 columns, respectively. All substrates were used at 100 mg/mL of GGM. The quantification of mannan-oligosaccharides (in terms of monomer equivalents) and monosaccharides were compared to the monomer equivalents of polymeric mannan, galactan and glucan and the degree of conversion was reported as % conversion. The standard deviations were within < 10 % in all cases. The same data as in Figure S5.
